# Supplementary material for: Point-of-care ultrasound reveals extensive pathology in Gabonese preschool-age children with urogenital schistosomiasis
Source: PLoS Negl Trop Dis. 2025 Apr 10;19(4):e0012991. doi: 10.1371/journal.pntd.0012991 (PMC12052205; doi:10.1371/journal.pntd.0012991)
Supplement: Attachment — Submitted filename: Response to reviewers.docx [file pntd.0012991.s003.docx]

Response to the editors and reviewers

**Journal Requirements:**

1) Please ensure that the CRediT author contributions listed for every co-author are completed accurately and in full.
At this stage, the following Authors/Authors require contributions: J. Remppis, A. Verheyden, A. Sultanli, A.L. Bustinduy, T. Heller, A.A. Adegnika, M.P. Grobusch, M. Ramharter, E. Joekes, and S. Bélard. Please ensure that the full contributions of each author are acknowledged in the "Add/Edit/Remove Authors" section of our submission form.
The list of CRediT author contributions may be found here: https://journals.plos.org/plosntds/s/authorship#loc-author-contributions

*Reply: The author contributions have been added to the submission form.*

2) Please upload all main figures as separate Figure files in .tif or .eps format. For more information about how to convert and format your figure files please see our guidelines: 
<https://journals.plos.org/plosntds/s/figures>

*Reply: All figures have been converted into .tif format and uploaded as separate files. The Preflight Analysis and Conversion Engine (PACE) digital diagnostic tool was used to ensure that all figures meet the PLOS requirements.*

3) We have noticed that you have uploaded Supporting Information files, but you have not included a list of legends. Please add a full list of legends for your Supporting Information files after the references list.

*Reply: The list of legends has been added at the end of the manuscript.*

4) We note that there is identifying data in the Supporting Information file(s) Database.xlsx. Prior to sharing human research participant data, authors should consult with an ethics committee to ensure data are shared in accordance with participant consent and all applicable local laws.
Data sharing should never compromise participant privacy. It is therefore not appropriate to publicly share personally identifiable data on human research participants. The following are examples of data that should not be shared:
 - Name, initials, physical address
 - Ages more specific than whole numbers
 - Internet protocol (IP) address
 - Specific dates (birth dates, death dates, examination dates, etc.)
 - Contact information such as phone number or email address
 - Location data
 - ID numbers that seem specific (long numbers, include initials, titled “Hospital ID”) rather than random (small numbers in numerical order)
Data that are not directly identifying may also be inappropriate to share, as in combination they can become identifying. For example, data collected from a small group of participants, vulnerable populations, or private groups should not be shared if they involve indirect identifiers (such as sex, ethnicity, location, etc.) that may risk the identification of study participants.
Additional guidance on preparing raw data for publication can be found in our Data Policy: https://journals.plos.org/plosntds/s/data-availability#loc-human-research-participant-data-and-other-sensitive-data
You may also find guidance in the following article: <http://www.bmj.com/content/340/bmj.c181.long>.
Please remove or anonymize all personal information: Specific dates, and ID numbers that seem specific, ensure that the data shared are in accordance with participant consent, and re-upload a fully anonymized data set. Please note that spreadsheet columns with personal information must be removed and not hidden as all hidden columns will appear in the published file.

*Reply: All personal information in the supporting information file Database.xlsx has been removed or anonymized.*

**Reviewers' comments:**

Reviewer's Responses to Questions

**Key Review Criteria Required for Acceptance?**

As you describe the new analyses required for acceptance, please consider the following:

**Methods**

-Are the objectives of the study clearly articulated with a clear testable hypothesis stated?

-Is the study design appropriate to address the stated objectives?

-Is the population clearly described and appropriate for the hypothesis being tested?

-Is the sample size sufficient to ensure adequate power to address the hypothesis being tested?

-Were correct statistical analysis used to support conclusions?

-Are there concerns about ethical or regulatory requirements being met?

Reviewer #1: All data are clearly presented

Reviewer #2: Are the objectives of the study clearly articulated with a clear testable hypothesis stated?
Yes.
-Is the study design appropriate to address the stated objectives?
Yes.
-Is the population clearly described and appropriate for the hypothesis being tested?
Yes.
-Is the sample size sufficient to ensure adequate power to address the hypothesis being tested?
Yes.
-Were correct statistical analysis used to support conclusions?
Yes.
-Are there concerns about ethical or regulatory requirements being met?
Yes.

Reviewer #3: The paper present a simple objective and followed good procedure. However, the procedure need expansion:

*Reply:*

*We thank the reviewer for his/her comment. We assume that the comment refers to the relatively brief description of our key study procedure, the point-of-care ultrasound protocol FASUS, in the methods section of our manuscript.*

*We agree that more detailed information about the ultrasound protocol is necessary to correctly contextualize our study results. An in-depth description and evaluation of the protocol and the associated training of ultrasound operators has been previously and separately published (Remppis J, Verheyden A, Bustinduy AL, Heller T, Garcia-Tardon N, Manouana GP, et al. Focused Assessment with Sonography for Urinary Schistosomiasis (FASUS)-pilot evaluation of a simple point-of-care ultrasound protocol and short training program for detecting urinary tract morbidity in highly endemic settings. Trans R Soc Trop Med Hyg. 2020;114(1):38-48 - Reference number 9 in our manuscript.)*

*Our manuscript primarily focuses on the detected pathology, rather than the detection method. We therefore prefer not to expand the methods section by repeating the details of the previously published ultrasound procedure.*

*If we misunderstood the comment, we are happy to further elaborate on the study procedures. In this case, please provide more precise information on which parts of the methods section need to be expanded.*

**Results**

-Does the analysis presented match the analysis plan?

-Are the results clearly and completely presented?

-Are the figures (Tables, Images) of sufficient quality for clarity?

Reviewer #1: Yes

Reviewer #2: Does the analysis presented match the analysis plan?
Yes.
-Are the results clearly and completely presented?
Yes.
-Are the figures (Tables, Images) of sufficient quality for clarity?
Yes.

Reviewer #3: The result is clearly stated

**Conclusions**

-Are the conclusions supported by the data presented?

-Are the limitations of analysis clearly described?

-Do the authors discuss how these data can be helpful to advance our understanding of the topic under study?

-Is public health relevance addressed?

Reviewer #1: Yes, definitely. Very important conclusions for improvement of control of schistosmiasis

Reviewer #2: -Are the conclusions supported by the data presented?
Yes.
-Are the limitations of analysis clearly described?
Yes.
-Do the authors discuss how these data can be helpful to advance our understanding of the topic under study?
Yes.
-Is public health relevance addressed?
Yes.

Reviewer #3: Fair

**Editorial and Data Presentation Modifications?**

Use this section for editorial suggestions as well as relatively minor modifications of existing data that would enhance clarity. If the only modifications needed are minor and/or editorial, you may wish to recommend “Minor Revision” or “Accept”.

Reviewer #1: None

Reviewer #2: Accept

Reviewer #3: sentences in line 213 is not clear to me.

*Reply:*

*The reviewer refers to the sentences “Of note, WHO recommendations valid prior and during the study period had not included PSAC in mass drug administration (MDA) programs. These recommendations have changed in the meantime due to increasing evidence that chronic pathology already affects children before school-age”.*

*We added these sentences to the manuscript because WHO recommendations on the prevention and treatment of schistosomiasis have changed between the time of study conduction and the time the manuscript was written:*

*In 2001, the WHO published its resolution WHA54.19 on schistosomiasis and soil-transmitted* *helminth infections. The respective recommendations on preventive chemotherapy interventions targeted on school-age children (aged 5–14 years) in endemic areas, as this age group was considered as having the highest risk of infection.*

*In 2012, the WHO released a progress report including a strategic plan for the years 2012 – 2020. One strategic action was to “assess the role played by preschool-age children in sustaining transmission of schistosomiasis and the need to extend treatment to this group; develop appropriate guidance on how to treat them”. However, the general recommendation by that time was that children aged under 4 years should “be treated on an individual basis by medical personnel”. Thus, during our study period, which was from December 2015 to June 2016, preventive chemotherapy was not generally recommended for preschool-age children.*

*It was only in 2022 that the WHO changed its recommendations: According to the current guideline on control and elimination of human schistosomiasis (Reference number 15 in our manuscript), annual preventive chemotherapy is now recommended for “all age groups from 2 years old” in endemic communities with prevalence of Schistosoma spp. infection ≥ 10%. The expansion of target age groups is based on several systematic reviews and meta-analysis.*

*Action taken:*

*In order to clarify the different recommendations, their chronological order, and the scientific background, we revised the section in our manuscript and added several references (References number 11 – 14).*

*Revised section: “Of note, by the years 2015 - 2016, when our study was conducted, the WHO did not generally recommend preventive chemotherapy for PSAC: WHO recommendations from 2001 targeted on SAC in endemic areas, and in a progress report released in 2012, a possible age extension to PSAC was discussed but not generally recommended [11, 12]. It was only 2022 that the WHO recommended preventive chemotherapy for children from two years in endemic communities, due to increasing evidence that chronic pathology already affects children before school-age [13 – 15].”*

**Summary and General Comments**

Use this section to provide overall comments, discuss strengths/weaknesses of the study, novelty, significance, general execution and scholarship. You may also include additional comments for the author, including concerns about dual publication, research ethics, or publication ethics. If requesting major revision, please articulate the new experiments that are needed.

Reviewer #1: Well written article with important epidemiologic and clinical significance

Reviewer #2: This study aimed to detect urinary tract pathology in symptomatic UGS patients in an endemic area and to correlate the findings with demographic, clinical, and parasitological factors. The authors reported the urinary tract pathology related to UGS in Gabon for the first time. This study has a chance to primarily guide local schistosomiasis control and can add valuable information to control strategies and individual patient care on a global level. Scientific sound of text and references are adequate.

Reviewer #3: The paper present a simple method in the work using known tool to test for S. hematobium among children. However, there was no any novelty in the work beyond looking at underage children. There should be additional study using more tools e.g. molecular diagnostics, RDT to further validate the claim of 'potentially irreversible pathology' among children. for an infection to be irreversible, it mean that genetic alteration had occurred and no evidence that such had taken place. Also, the result is too little for publication except case report

*Reply:*

*We thank the reviewer for his/her comment. We agree that ultrasound is a well-established tool in the detection of UGS-related pathology, including children. However, as the reviewer already stated, few studies have used ultrasound in cohorts of preschool-children symptomatic of UGS. Furthermore, our study is the first to report such pathology into detail from Central Africa. For these reasons, we believe that our study adds interesting new insights into the disease manifestation and provides additional evidence to include preschool-children into preventive strategies.*

*Regarding the reviewer’s second comment, we understand that genetic alteration is a prerequisite for irreversibility in some instances. However, in our manuscript we do not claim* S. haematobium *infection to be potentially irreversible, but rather the associated pathology. In the case of urogenital schistosomiasis, potentially irreversible pathology (i.e. ureteral obstruction, hydronephrosis) of the urinary tract is a consequence of chronic inflammation, and consecutive fibrosis of the bladder and ureter walls; this does not necessarily require persistent infection or presence of parasites. Urinary tract pathology can still be detected in some patients who previously had* S. haematobium *infection, but have been successfully treated. These persistent pathologies can cause substantial morbidity, especially if they occur early in life. In our case, irreversible pathology may be seen as persistent “scars” even after successful treatment of the etiologic infection.*

*The best means to detect UGS-related pathologic changes of the urinary tract is by imaging, i.e. ultrasound, MRI or cystoscopy. Rapid diagnostic or molecular tests, as suggested by the reviewer, are helpful tools in the detection of* S. haematobium *infection; but cannot replace imaging in terms of morbidity assessment.*
